# Pathway-based metabolomics study of sarcopenia-related traits in two US cohorts

**DOI:** 10.18632/aging.203926

**Published:** 2022-03-02

**Authors:** Qi Zhao, Hui Shen, Jiawang Liu, Chi-Yang Chiu, Kuan-Jui Su, Qing Tian, David Kakhniashvili, Chuan Qiu, Lan-Juan Zhao, Zhe Luo, Hong-Wen Deng

**Affiliations:** 1 Department of Preventive Medicine, College of Medicine, University of Tennessee Health Science Center, Memphis, TN 38163, USA; 2 Tulane Center for Biomedical Informatics and Genomics, Deming Department of Medicine, School of Medicine, Tulane University, New Orleans, LA 70112, USA; 3 Medicinal Chemistry Core, Office of Research, University of Tennessee Health Science Center, Memphis, TN 38163, USA; 4 Department of Pharmaceutical Science, College of Pharmacy, University of Tennessee Health Science Center, Memphis, TN 38163, USA; 5 Proteomics and Metabolomics Core, Office of Research, University of Tennessee Health Science Center, Memphis, TN 38163, USA

**Keywords:** grip strength, metabolite, metabolomics, muscle mass, sarcopenia

## Abstract

We aimed to validate two metabolites, aspartic acid and glutamic acid, which were associated with sarcopenia-related traits, muscle mass and strength, in our previous untargeted metabolomics study and to identify novel metabolites from five metabolic pathways involving these two metabolites. We included a discovery cohort of 136 white women aged 20-40 years (used for the previous untargeted metabolomics analysis) and a validation cohort of 174 subjects aged ≥ 60 years, including men and women of white and black. A targeted LC-MS assay successfully detected 12 important metabolites from these pathways. Aspartic acid was associated with muscle mass and strength in the discovery cohort, but not in the validation cohort. However, glutamic acid was associated with these sarcopenia traits in both cohorts. Additionally, N-acetyl-L-aspartic acid and carnosine were the newly identified metabolites that were associated with muscle strength in the discovery and validation cohorts, respectively. We did not observe any significant sex and race differences in the associations of these metabolites with sarcopenia traits in the validation cohort. Our findings indicated that glutamic acid might be consistently associated with sarcopenia-related traits across age, sex, and race. They also suggested that age-specific metabolites and metabolic pathways might be involved in muscle regulation.

## INTRODUCTION

Sarcopenia, characterized by the age-related loss of muscle mass and strength, is a major contributor to the risk of physical frailty, loss of independence, and hospitalization with poor health outcomes in aging older adults [1]. It has become a major public health challenge with the rapid expansion of the world’s older population and is responsible for substantial healthcare expenditure [2]. Sarcopenia was recognized as an independent condition with an assigned ICD-10 code in 2016, which is a milestone for the increased awareness of its importance in human health [3]. Age-related sarcopenia is a multifactorial disease, and some possible causes have been suggested, such as decreased nerve input, protein intake, and physical activity [4–7]. However, the biological mechanisms underlying the development of sarcopenia are still largely unknown, and no specific drugs have been approved for the treatment of sarcopenia [1].

Metabolomics is an emerging technology to comprehensively profile small molecules in biofluids, cells, and tissues and has contributed to our understanding of a number of disorders [8–11], such as diabetes, cardiovascular disease, and cancer. Metabolomics methodologies fall into two distinct groups, untargeted and targeted metabolomics, each with its own inherent advantages and disadvantages. Untargeted metabolomics is the comprehensive analysis of all the measurable analytes in a sample. It is especially suitable for diseases/conditions with very limited information about its metabolic mechanisms. By contrast, targeted metabolomics is the measurement of defined groups of chemically characterized and biochemically annotated metabolites [12]. Although most studies have used either the untargeted or targeted approach, it is reasonable, or even ideal, to use the untargeted metabolomics approach to prioritize metabolic pathways (hypothesis generation) for further comprehensive targeted metabolomics analysis in additional samples which can validate findings of the untargeted analysis and discover more disease-related metabolic changes.

To investigate the mechanisms of sarcopenia, we have conducted an untargeted metabolomics study of sarcopenia-related traits, muscle mass and strength, in a cohort of young white women. In the pathway analysis, two amino acids, aspartic acid and glutamic acid, were both mapped to five metabolic pathways [13]. To further validate the associations of these two metabolites with muscle mass (measured by body mass index-adjusted appendicular lean mass, ALM/BMI) and muscle strength (measured by hand grip strength, HGS) and identify additional trait-associated metabolites, we designed a targeted metabolomics assay to assess the key metabolites from these pathways in the original discovery cohort of white young women and a validation cohort including older black and white as well as men and women.

## RESULTS

The clinical characteristics of the study participants in the discovery and validation cohorts are shown in Table 1. Across all the sex and race subgroups, the participants of the validation cohort were older, had a higher rate of current smoking, and had less alcohol drinking compared to those of the discovery cohort. The validation cohort had similar levels of ALM/BMI but smaller hand grip strength, especially among white and black women, than the discovery cohort.

**Table 1:** Characteristics of study participants.

	**Discovery cohort**	**Validation cohort**				
		**Overall**	**White women**	**White men**	**Black women**	**Black men**
N	136	174	71	31	19	53
Age, year	31.5 (5.1)	65.9 (4.7)	67.7 (5.0)	67.1 (5.1)	64.6 (3.2)	63.4 (2.8)
Weight, kg	70.3 (21.4)	78.6 (38.9)	71.9 (56.8)	87.6 (15.7)	77.5 (19.7)	82.6 (17.1)
Height, cm	164.6 (6.4)	168.8 (9.5)	162.3 (5.9)	175.3 (7.8)	163.1 (5.5)	175.6 (8.3)
Body mass index, kg/m^2^	26.0 (7.5)	27.6 (14.4)	27.3 (21.5)	28.6 (5.5)	29.1 (7.2)	26.8 (5.3)
Current smoking, %	35.3	56.9	43.7	54.8	47.4	79.2
Alcohol drinking, gram/day	36.5 (52.4)	12.3 (24.2)	10.3 (14.3)	9.1 (10.5)	14.6 (43.4)	16.0 (30.3)
Physical activity, times/week	3.1 (2.2)	3.5 (2.6)	3.7 (2.4)	3.1 (2.6)	2.6 (2.4)	3.8 (3.0)
Dairy intake, cups/day	1.6 (1.3)	1.6 (1.4)	1.8 (1.6)	1.8 (1.5)	1.3 (1.0)	1.3 (1.1)
ALM/BMI index	0.8 (0.1)	0.8 (0.2)	0.7 (0.1)	0.9 (0.1)	0.7 (0.1)	1.0 (0.2)
Hand grip strength, kg	27.0 (8.4)	23.6 (9.5)	17.8 (4.5)	31.0 (10.0)	15.4 (4.3)	29.8 (8.1)

Means (standard deviations) are for continuous variables, and percentages are for categorical variables.

ALM/BMI, body mass index adjusted appendicular lean mass.

**Figure 1:**
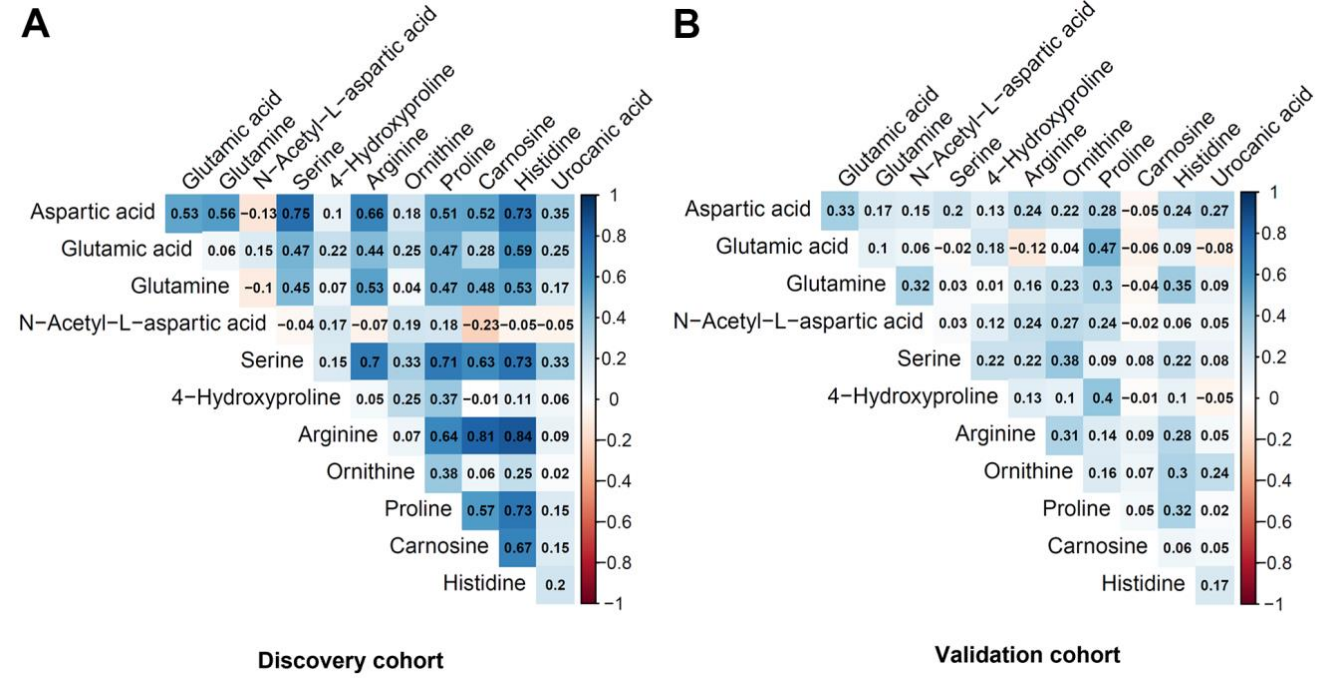
**Pairwise correlation coefficients among the metabolites in the targeted assay.** (**A**) Discovery cohort. (**B**) Validation cohort.

**Figure 2:**
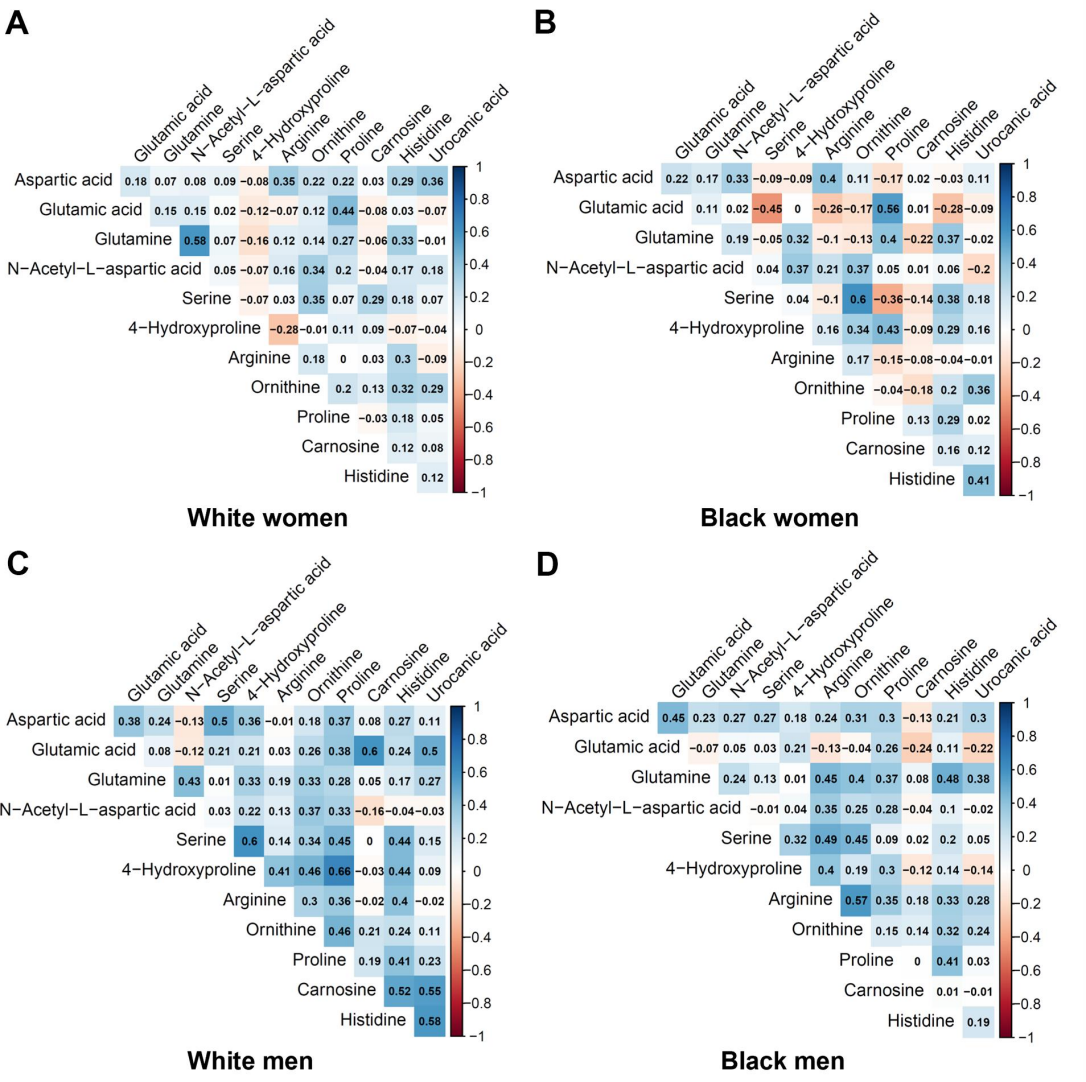
**Pairwise correlation coefficients among the metabolites in the race-sex subgroups of the validation cohort.** (**A**) White women. (**B**) Black women. (**C**) White men. (**D**) Black men.

Table 2 shows the metabolites selected and measured from the five metabolic pathways involving aspartic acid and glutamic acid. Twelve out of the 15 metabolites on the targeted liquid chromatography-mass spectrometry (LC-MS) assay were successfully detected in the study samples. Figure 1 shows the correlation coefficients between the 12 detected metabolites in the discovery and validation cohorts, respectively. In general, the correlations among the metabolites were very different between the subjects in the discovery cohort and the validation cohort. Additionally, the correlations of metabolites varied substantially among different race- and sex-subgroups as well (Figure 2). These different correlations of metabolites within each metabolic pathway among different age, race, and sex groups may suggest the functions of the pathways probably vary across age, sex, and race as well.

**Table 2:** Selected metabolites from the metabolic pathways involving aspartic acid and glutamic acid.

**Metabolic pathway**	**Metabolites**
Alanine, aspartate, and glutamate metabolism	Aspartic acid, Glutamic acid, N-Acetyl-L-aspartic acid, Glutamine, *Oxaloacetate, γ-Aminobutanoate*
Aminoacyl-tRNA biosynthesis	Aspartic acid, Glutamic acid, Serine
Arginine and proline metabolism	Aspartic acid, Glutamic acid, Arginine, Ornithine, Proline, 4-Hydroxyproline
Histidine Metabolism	Aspartic acid, Glutamic acid, Histidine, Urocanic acid, Carnosine, *Histamine*
Nitrogen metabolism	Aspartic acid, Glutamic acid

The metabolites in italic were not detected in the study samples.

The multivariate analysis showed that aspartic acid, identified in our previous untargeted metabolomics study, was still significantly associated with sarcopenia traits in the discovery cohort, but not in the validation cohort (Table 3). Higher aspartic acid levels were associated with lower ALM/BMI and HGS (Table 4). However, glutamic acid, another metabolite identified in our previous untargeted metabolomics study, was significantly associated with sarcopenia traits in both the discovery and validation cohorts even after adjusting for multiple testing (FDR q-value < 0.05) (Table 3). Specifically, glutamic acid was associated with ALM/BMI (*P* = 0.011) in the discovery cohort but associated with both ALM/BMI (*P* = 0.024) and HGS (*P* = 0.001) in the validation cohort. Glutamic acid was negatively associated with ALM/BMI in both the discovery and validation cohorts and negatively associated with HGS in the validation cohort (Table 4). We did not observe any race- or sex-specific effects of glutamic acid on the sarcopenia traits in the validation cohort (*P* values for the interaction terms of the metabolite with race and sex > 0.05) (Table 3).

**Table 3:** The significance of the associations between metabolites and sarcopenia traits in the joint and individual trait analyses.

**Metabolite**	**Class**	**HMDB ID**	**Discovery cohort**				**Validation cohort**				
			**Joint analysis**	**ALM/BMI**	**HGS**		**Joint** **analysis**	**Interaction with race**	**Interaction with sex**	**ALM/BMI**	**HGS**
Aspartic acid	Amino acid	HMDB0000191	0.009*	**0.023**	**0.010**		0.917	0.539	0.588	0.880	0.704
Glutamic acid	Amino acid	HMDB0000148	0.025*	**0.011**	0.132		0.001*	0.428	0.259	**0.024**	**0.001**
Glutamine	Amino acid	HMDB0000641	0.466	0.585	0.409		0.623	0.080	0.335	0.347	0.876
N-Acetyl-L-aspartic acid	Amino acid	HMDB0000812	0.006*	0.178	**0.002**		0.682	0.102	**0.046**	0.548	0.471
Serine	Amino acid	HMDB0000187	0.964	0.920	0.842		0.214	0.668	0.500	0.288	0.294
4-Hydroxyproline	Amino acid	HMDB0000725	0.762	0.475	0.803		0.929	0.703	0.292	0.714	0.868
Arginine	Amino acid	HMDB0000517	0.679	0.582	0.677		0.957	0.293	0.995	0.959	0.796
Ornithine	L-alpha-amino acid	HMDB0000214	0.393	0.199	0.874		0.934	0.624	0.708	0.726	0.861
Proline	Amino acid	HMDB0000162	0.357	0.870	0.176		0.492	0.462	0.319	0.329	0.416
Carnosine	Hybrid peptide	HMDB0000033	0.485	0.292	0.924		**0.043**	0.060	**0.022**	0.411	**0.038**
Histidine	Amino acid	HMDB0000177	0.391	0.186	0.567		0.164	0.471	0.657	0.589	0.117
Urocanic acid	Imidazole	HMDB0000301	0.667	0.952	0.406		0.958	0.443	0.526	0.942	0.828

*FDR q-value < 0.05. Raw *P* values < 0.05 are bold.

ALM/BMI, body mass index adjusted appendicular lean mass; HGS, hand grip strength; HMDB, the Human Metabolome Database.

**Table 4:** The effects associated with sarcopenia traits in the discovery and validation cohorts.

**Metabolite**	**ALM/BMI**							**HGS**					
	**Discovery cohort**	**Validation cohort**						**Discovery cohort**	**Validation cohort**				
		**Overall**	**White**	**Black**	**Women**	**Men**			**overall**	**White**	**Black**	**Women**	**Men**
Aspartic acid	-0.0217	-0.0016	-0.0122	0.0251	-0.0185	0.0061		-1.8126	-0.2053	-0.0213	-0.5099	-0.1444	-0.4212
Glutamic acid	-0.0242	-0.0255	-0.0219	-0.0159	-0.0217	-0.0171		-1.0832	-1.8610	-2.7074	-1.4658	-1.6002	-2.8643
Glutamine	0.0055	0.0096	0.0051	0.0242	0.0114	0.0020		-0.6150	0.0828	0.6324	-0.9101	-0.3811	0.4758
N-Acetyl-L-aspartic acid	0.0132	0.0062	-0.0006	0.0067	-0.0031	0.0035		2.2071	0.3889	0.9694	-0.1936	-0.7651	0.7507
Serine	-0.0010	0.0111	0.0066	0.0134	0.0087	0.0083		0.1427	-0.5708	-0.8434	0.0111	-0.0173	-0.6966
4-Hydroxyproline	0.0071	-0.0040	0.0096	-0.0102	0.0239	-0.0175		0.1824	-0.0942	0.0443	-0.6128	0.8805	-0.8552
Arginine	-0.0053	-0.0005	-0.0160	0.0163	-0.0057	0.0027		0.2956	-0.1371	-0.4751	0.5423	-0.1930	0.0777
Ornithine	0.0126	0.0037	-0.0059	0.0107	-0.0012	0.0031		0.1149	0.0960	-0.1391	0.0735	-0.1766	0.3235
Proline	0.0016	-0.0110	-0.0157	0.0068	-0.0171	-0.0040		0.9811	-0.4799	-0.0328	-0.7278	0.1212	-0.8645
Carnosine	-0.0103	0.0083	0.0117	0.0036	0.0119	0.0026		0.0681	-1.0917	-1.8010	-0.7936	0.3106	-1.5213
Histidine	-0.0130	0.0056	-0.0027	0.0194	-0.0080	0.0101		-0.4155	-0.8430	-0.6724	-1.2067	-0.2238	-1.3029
Urocanic acid	-0.0006	0.0007	-0.0141	0.0199	-0.0168	0.0210		-0.5849	-0.1167	-0.7393	1.0734	-0.5771	0.9171

All the effects were associated with 1-standard deviation changes in metabolites.

ALM/BMI, body mass index adjusted appendicular lean mass; HGS, hand grip strength.

N-Acetyl-L-aspartic acid (NAA) and carnosine were two potential cohort-specific metabolites associated with HGS. NAA was significantly associated with HGS in the discovery cohort (*P* for joint analysis = 0.006; effect = 2.2071 and *P* = 0.002 for HGS), and carnosine was suggestively associated with HGS in the validation cohort (*P* for joint analysis = 0.043; effect = -1.0917 and *P* = 0.038 for HGS) (Tables 3, 4). There were also suggestive (not significant after adjusting for the multiple testing) sex-specific effects of both NAA (*P* for interaction with sex = 0.046) and carnosine (*P* for interaction with sex = 0.022) on the sarcopenia traits in the validation cohort (Table 3). Especially, the effects on HGS were opposite in women and men for both N-acetyl-L-aspartic acid and carnosine (Table 4).

## DISCUSSION

This targeted metabolomics study replicated the study findings of a previous untargeted metabolomics study, the association of glutamic acid with sarcopenia traits, not only in the original discovery cohort (for the previous untargeted metabolomics study) but also in another independent validation cohort. Although we did not replicate the association of aspartic acid, which was another metabolite identified in our previous untargeted metabolomics study, with sarcopenia traits in the validation cohort, we did replicate it in the discovery cohort using the targeted metabolomics method. Additionally, we identified two novel metabolites, NAA and carnosine, which were potentially associated with the sarcopenia trait, muscle strength, by measuring more metabolites from the metabolic pathways involving glutamic acid and aspartic acid.

Although untargeted metabolomics analysis has its advantages in discovering novel biomarkers without the need for a priori metabolic hypothesis over targeted metabolomics analysis, it still has many challenges or pitfalls, especially compound identification [14, 15]. In addition to a large proportion of unknown metabolites, the structures of metabolites identified through existing MS databases still need further verification through comparing with authentic standards tested using the same instrument as study samples, a “gold standard” for determining metabolite identities [16]. Also, the structure verification of disease-related metabolites is necessary for subsequent functional studies for the ultimate translational goal of metabolomics study findings. Therefore, targeted metabolomics analysis designed using chemical standards of metabolites identified in untargeted metabolomics studies will help to further validate the metabolite identities and their associations with traits/diseases. In this study, we also included additional metabolites from the disease-related metabolic pathways found in the previous untargeted metabolomics study in the targeted metabolomics assay to further identify novel biomarkers for sarcopenia traits.

In this study, we replicated the associations of aspartic acid and glutamic acid with sarcopenia traits in the discovery cohort of young white women using the new targeted LC-MS assay. Glutamic acid was also associated with sarcopenia traits in the new validation cohort of older subjects, including both black and white and men and women. These findings might suggest glutamic acid influences these sarcopenia traits across age, sex, and race. However, aspartic acid might be a sarcopenia trait-related metabolite that is specific to younger individuals. Aspartic acid and glutamic acid are not essential amino acids but among the 20 proteinogenic amino acids. Aspartic acid can be made from glutamic acid by enzymes using vitamin B6. Both aspartate and glutamate (the anions of the amino acids) are major excitatory neurotransmitters [17], and glutamate also plays roles in the skeletal neuromuscular junction. Participating in modulating cholinergic transmission and plastic changes [18]. Glutamate in skeletal muscle also participates in various metabolic pathways, such as glutathione synthesis, insulin production, tricarboxylic acid cycle, and purine nucleotide cycle [19]. Additionally, glutamic acid can be converted to γ-aminobutyric acid (GABA) by the enzyme glutamic acid decarboxylase. GABA is the most abundant inhibitory transmitter in the brain. Oral supplementation of GABA has been reported to increase growth hormone and muscle protein synthesis, potentially contributing to dynamic protein turnover [20]. The association of glutamic acid with muscle mass we identified was in line with the finding in a sample of UK women [21]. However, we added further evidence for its association with muscle strength in humans as well. In addition, animal studies have shown that aspartate inhibits inflammation-induced muscle loss through regulating phosphorylation of Akt, AMPKα, and FOXO1 [22, 23]. However, we identified aspartate were negatively associated with muscle mass and strength in humans. These inconsistent findings warrant more studies to clarify its role in muscle regulation.

NAA is a derivative of aspartic acid and the second most concentrated molecule in the brain after the amino acid glutamic acid. NAA is essential for normal brain operation and declines in several neurodegenerative and neuropsychiatric diseases [24]. Also, NAA is reduced in the aging spinal cord that contributes to loss of innervation and downstream degenerative processes that occur in skeletal muscle [25]. NAA is synthesized in neuronal mitochondria but can efflux from the brain to the circulation. It has been observed that serum levels of NAA decreased with aging [26], suggesting its effects on health conditions/diseases might vary with age. This might partially explain why it was associated with muscle strength only in the discovery cohort of young subjects, but not in the validation cohort of older subjects in this study.

Carnosine is a dipeptide of the amino acids beta-alanine and histidine with a high concentration in mammalian skeletal muscle. In skeletal muscle cells, carnosine can be synthesized by carnosine synthase from beta-alanine and histidine. Interestingly, muscle carnosine loading leads to improved performance in high-intensity exercise in both untrained and trained individuals [27]. Also, carnosine has the potential to suppress many of the biochemical changes that accompany aging, such as protein oxidation, glycation, and cross-linking, and associated pathologies [28]. In this study, we observed a suggestive sex difference in the effects of plasma carnosine levels on muscle strength among older subjects. Carnosine was positively associated with muscle strength in women, but negatively in men. Blood carnosine is partially influenced by dietary factors, such as intakes of carnosine and beta-alanine [29]. Therefore, reverse causation might be an explanation for the negative association between carnosine and muscle strength in men. For example, carnosine supplementation was taken among the elderly with reduced muscle function.

Our study has several strengths. First, we implemented a novel strategy of metabolomics study, which was to conduct targeted metabolomics analysis following up the study findings of an untargeted metabolomics study. Second, a pathway-based approach was used in the targeted metabolomics analysis to further identify sarcopenia trait-related metabolites. Third, two diverse study cohorts were included to identify common and specific outcome-related metabolites for different age, race, and sex subgroups. Finally, we investigated two major sarcopenia traits, muscle mass and strength, at the same time instead of muscle mass only which most of the previous studies focused on. In fact, muscle strength is better than muscle mass in predicting adverse outcomes [30]. Nevertheless, our study also has some limitations. The sample size for each race-sex subgroup in the validation cohort was relatively small, especially for white men and black women, which might have limited the study power for examining the potential race and sex differences in metabolites associated with sarcopenia traits. Although we have used comprehensive pathway analysis to select metabolites from the metabolic pathways of interest for the targeted metabolomics analysis, it is still possible that we have missed some important metabolites that were not tested in the targeted LC-MS assay.

In conclusion, this targeted metabolomics study replicated some findings from the previous untargeted metabolomics study of sarcopenia traits and also identified novel metabolites associated with sarcopenia traits. Our findings suggest that glutamic acid might be a risk factor for sarcopenia traits across all age, sex, and race groups. However, the effects of aspartic acid, NAA, and carnosine might vary with age. The biological mechanisms underlying the relationship between these metabolites and sarcopenia traits still need to be clarified in future studies.

## MATERIALS AND METHODS

### Study subjects

We included two study cohorts, the discovery and validation cohorts. The discovery cohort consisted of 136 white women aged 20-40 years from the ongoing Louisiana Osteoporosis Study (LOS), which aims to build a large sample pool and database for investigating genetic and environmental factors for osteoporosis in Southern Louisiana. The inclusion and exclusion criteria of LOS have been described in our previous study [31]. Individuals who were pregnant, had a bilateral oophorectomy, or had any chronic conditions (such as diabetes mellitus, renal failure, liver failure, lung disease, gastrointestinal disease, and inherited bone disease) were excluded from the current study. The validation cohort was from the ongoing MetAbolomics Study of Sarcopenia (MASS), which was designed to conduct metabolomic profiling of sarcopenia traits among individuals ≥ 60 years from the New Orleans metropolitan area, Louisiana. In this study, we included 174 sequential participants recruited in MASS till conducting this study (71 white women, 31 white men, 19 black women, and 53 black men). The exclusion criteria of MASS include 1) prolonged bed rest caused by any reasons; 2) chronic failure of heart, lung, liver, and kidney; 3) nervous system diseases (e.g., stroke, spinal cord injuries, and dementia); 4) uncontrolled diabetes mellitus; 5) chronic lung/gastrointestinal diseases/cancers; 6) severe rheumatoid arthritis; 7) active cancer treatment in the last years or cancer cachexia; 8) any type of congenital muscular dystrophy or metabolic disorders; and 9) alcohol or other substance abuse. Both LOS and MASS were approved by the institutional review board, and a written consent form was signed by each participant before any data and biosample collection.

### Clinical measurements

The participants of the two cohorts completed an interviewer-assisted comprehensive questionnaire to collect demographic information, lifestyle (including smoking, drinking, and physical activity), dietary factors (including dairy consumption), reproductive and medical history [13]. Weight was measured in light indoor clothing using a calibrated balance beam scale, and height was measured using a calibrated stadiometer without shoes. Body mass index (BMI) was calculated as weight (kg) divided by height squared (m^2^).

In both cohorts, total body and regional measures of lean mass were acquired using a dual-energy X-ray absorptiometry machine (Hologic Inc., Bedford, MA, USA) by trained and certified research staff. The machine was calibrated daily, and software and hardware were kept up to date during the data collection process. ALM was calculated as the sum of lean mass in the arms and legs. The BMI-adjusted ALM (ALM/BMI) was used to assess individuals’ muscle mass in the study [13, 32]. HGS was measured using the Jamar 1 hand-held dynamometer (TEC Inc., Clifton, NJ, USA). Two measurements of strength were taken at both hands. The maximum grip strength value of the two hands was used to assess an individual’s muscle strength.

### Pathway-based metabolite selection

**Figure 3:**
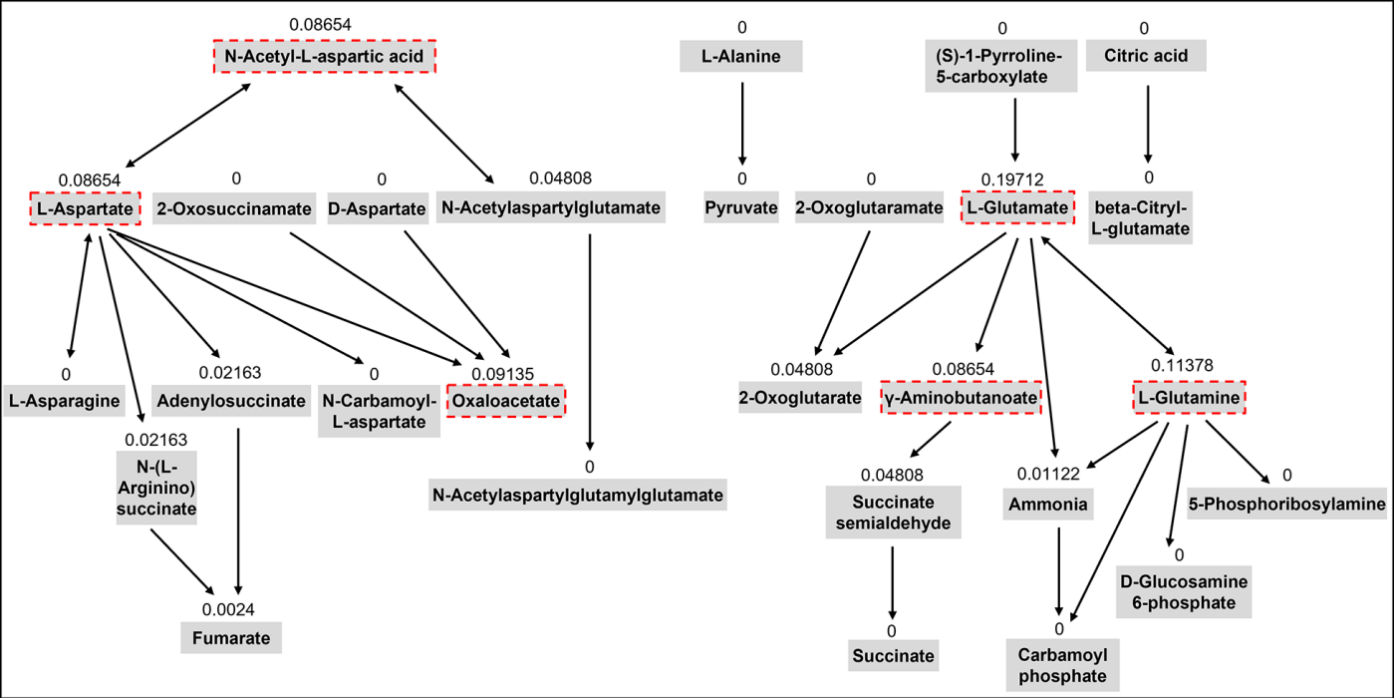
**The topology analysis of the pathway of alanine, aspartate, and glutamate metabolism.** The metabolites with red dash lines are the metabolites we selected from the pathway for the targeted assay. The number above each metabolite is the importance score of the metabolite in the pathway based on the topology analysis.

In our previous untargeted metabolomics study, we identified five metabolic pathways including aspartic acid and glutamic acid which were significantly associated with sarcopenia traits using the pathway enrichment analysis integrated into the MetaboAnalyst web tool [13]. This tool uses the pathway data from the KEGG database. Chemical compounds can be mapped to the pathways using HMDB IDs. This method also conducts topological analysis to assess the impact of each metabolite on the pathway of interest. It uses relative betweenness centrality and out-degree centrality measures of a metabolite in a pathway to calculate its importance, a score ranging from 0 to 1 [33]. In this study, we selected 15 metabolites from the five metabolic pathways involving aspartic acid and glutamic acid based on their importance scores over 0.05 in the topological analysis and chemical characteristics for the LC-MS analysis (Table 2). Figure 3 shows the topology analysis results of the alanine, aspartate, and glutamate metabolism pathway as an example to show how we selected the metabolites for the pathway.

### Targeted metabolomics analysis

### Sample preparation

Blood samples were collected from the study subjects after over 10 hours overnight fasting. Serum samples from the discovery cohort and plasma samples from the validation cohort were available and used for the metabolomics analysis. Serum or plasma samples were separated and stored in freezers at -80° C before this study. To each thawed serum or plasma sample (100 μL) in a 1.5 mL microcentrifuge tube, 20 μL of internal standard solution containing 4 μg/mL L-Proline-^13^C_5_,^15^N and 40 μg/mL L-Aspartic acid-2,3,3-d_3_ (Cambridge Isotope Laboratories, Inc., Tewksbury, MA, USA) was added, followed by vortex mixing for 20 second. Then, 800 μL of acetonitrile/acetone/methanol (8:1:1, v/v/v) was added for protein precipitation. After a stand still at -20° C for 20 min, and the mixture was centrifuged at 15,000 rpm for 10 min at -10° C. The supernatant (500 μL) was transferred to a new 1.5 mL microcentrifuge tube and dried under a gentle stream of nitrogen gas. The dried sample was reconstituted in 75 μL of 5% acetonitrile in water followed by centrifugation at 15,000 rpm for 5 min at -10° C. The supernatant (50 μL) was transferred into a 300 μL autosampler vial (Waters), placed at 4° C before the LC-MS/MS analysis.

### LC-MS/MS analysis for targeted profiling

The LC-MS/MS analysis of prepared samples was performed on SCIEX Triple Quad 5500 (AB Sciex LLC, Framingham, MA, USA) with Shimadzu Nexera XR HPLC (Shimadzu Scientific Instruments, Columbia, MA, USA) in the positive ionization mode. We used well-established HPLC/MS/MS methods to analyze the metabolites included in this study [34], with optimization of DP and CE on AB SCIEX 5500. Prepared test samples were placed in a SIL-20AC XR autosampler (Shimadzu Scientific Instruments, Columbia, MA, USA) that was set at 10° C. Chromatographic separation was achieved using an Acclaim™ 120 C18 column (2.1 × 100 mm, 3 μm; Thermo Fisher Scientific Inc., Waltham, MA, USA) maintained at 30° C with an injection volume of 1.0 μL. Mobile phase A consisted of 0.1% formic acid in water/acetonitrile (95:5, v/v) and mobile phase B was 0.1% formic acid in acetonitrile. The HPLC column was equilibrated with 100% mobile phase A at 30° C, and the linear gradient used for elution was 0% B from 0-1 min, 0-30% B from 1-3 min, 30-95% B from 3-3.5 min, 95-99% B from 3.5-4 min, 99% B from 4-5 min, 99-0% B from 5-5.5 min, and 0% B from 5.5-6 min. The total elution of a typical injection was 6.0 min at a flow rate of 0.25 mL/min, with a 2 min interval for column equilibration. The optimal MS/MS conditions for each metabolite were determined by individual standards (100 ng/mL in methanol) in the positive ionization mode. Retention times and MRM transitions of each metabolite are summarized in Supplementary Table 1. The blank control was prepared as 5% acetonitrile in water, and 5 μL of 20 random post-extracted plasma samples were pooled to form the QC sample. The blank control and QC sample were analyzed prior to the first tested sample and after every ten samples to monitor the instrument variability. The relative standard deviations of the analyzed metabolites were < 10%, showing good stability and reproducibility of the analytical system used in this study.

### LC-MS/MS data handling

Results collected from LC-MS/MS was analyzed in MultiQuart software 3.0.2 (AB Sciex LLC, Framingham, MA, USA). Analyte peaks in each MRM transition were automatically recognized in MultiQuart and checked manually. As internal standards were defined, the relative levels of each test metabolite were presented as the peak area ratio of the metabolite to the internal standard. Three of the 15 selected metabolites (oxalacetic acid, γ-aminobutyric acid, and histamine) were not detected in the samples using the targeted LC-MS method (Table 4). The other 12 metabolites had been detected in all the samples, and their abundance data were further log transformed and autoscaled to have zero mean and unit variance (z scores) using the R package ‘specmine’ [35].

### Statistical analyses

The characteristics of the participants were summarized using means (standard deviations) for continuous variables and percentages for categorical variables. Pairwise Pearson correlation coefficients were calculated to assess the relationship between metabolites in the discovery and validation cohorts, respectively. We used a multivariate analysis method to jointly examine the associations of metabolites with sarcopenia traits of interest, ALM/BMI and HGS, using the seemingly unrelated regression of the R package ‘systemfit’ [36]. The null hypothesis (H_0_) was that none of the traits was associated with the tested metabolite. At least one trait associated with the metabolite would reject the null hypothesis. The coefficients for each outcome variables were estimated. Potential confounding factors, including age, BMI (for HGS), smoking, alcohol drinking, physical activities, and dairy intakes, were adjusted in these models. For the validation cohort, race and sex were also adjusted. The interaction terms of race and metabolites as well as sex and metabolites were also included in these models to examine the race- and sex-specific effects of the metabolites on the sarcopenia traits in the validation cohort. Race- and sex-stratified analysis were also conducted to estimate the effects of each metabolite on the sarcopenia traits. The false discovery rate (FDR) method was used to adjust for multiple testing [37].

## Supplementary Material

Supplementary Table 1
