# Supplementary material for: Pathway-based metabolomics study of sarcopenia-related traits in two US cohorts
Source: Aging (Albany NY). 2022 Mar 2;14(5):2101–12. doi: 10.18632/aging.203926 (PMC8954970; doi:10.18632/aging.203926)
Supplement: Supplementary Table 1 [file aging-14-203926-s001.pdf]

SUPPLEMENTARY TABLE

Supplementary Table 1. Retention times and MRM transitions of metabolites quantified by LC-MS/MS.

Analyte	Ion transition (*m/z*)	Declustering potential (V)	Collision energy (eV)	Retention time (min)
Serine	106.00 > 60.00	10	21	1.19
Proline	116.00 > 70.00	60	21	1.25
L-Proline-$^{13}C_5$,^{15}N	122.10 > 75.00	60	21	1.25
4-Hydroxyproline	132.00 > 86.00	10	21	1.20
Ornithine	133.10 > 70.00	6	21	1.02
Aspartic acid	134.0 > 74.00	61	25	1.22
L-Aspartic acid-2,3,3-d_3	137.10 > 75.00	60	21	1.22
Urocanic acid	139.00 > 93.00	6	21	1.44
Glutamine	147.00 > 84.00	60	21	1.19
Glutamic acid	148.00 > 84.00	60	21	1.20
Histidine	156.00 > 110.00	6	21	1.03
Arginine	175.10 > 70.00	1	45	1.19
N-Acetyl-L-aspartic acid	176.10 > 134.00	31	15	1.59
Carnosine	227.10 > 110.00	41	37	0.99
